# Synergistic Chemical and Field-Effect Passivation Inhibits Sn^2+^ Oxidation and Non-Radiative Recombination in Tin–Lead Perovskite Solar Cells

**DOI:** 10.3390/ma19101914

**Published:** 2026-05-07

**Authors:** Jiahao Liu, Xucheng Wang, Pan Li, Huiyan Chen, Xing Tang, Weidong Lin, Ye Yuan, Xuehui Xu

**Affiliations:** 1 State Key Laboratory of Advanced Technology for Materials Synthesis and Processing, Wuhan University of Technology, Wuhan 430070, China; 345201@whut.edu.cn (J.L.); wxc322223@whut.edu.cn (X.W.); 345253@whut.edu.cn (X.T.); 359286@whut.edu.cn (W.L.); 2 State Key Laboratory of Modern Optical Instrumentation, Institute of Advanced Photonics, College of Optical Science and Engineering, Zhejiang University, Hangzhou 310027, China; 22430084@zju.edu.cn (P.L.); 12330062@zju.edu.cn (H.C.)

**Keywords:** perovskite solar cells, tin–lead perovskite solar cells, interface passivation, field-effect passivation

## Abstract

Narrow-bandgap tin–lead (Sn–Pb) perovskite solar cells (PSCs) are essential for high-performance tandem photovoltaics, yet their operational stability and efficiency suffer from spontaneous Sn^2+^ oxidation, interfacial defects, and non-radiative recombination. Current passivation strategies often provide only a single modification mode and struggle to adequately stabilize Sn^2+^ without introducing charge-transport barriers. Here, we introduce morpholine acetate (MPAC) as a novel interfacial passivator to achieve synergistic chemical and field-effect passivation in Sn–Pb perovskites. The acetate group of MPAC coordinates with undercoordinated metal cations, suppressing Sn^2+^ oxidation and minimizing defect states. Simultaneously, the morpholine moiety forms an interfacial dipole layer that aligns energy levels to facilitate charge extraction. Consequently, MPAC-modified PSCs achieve a champion power conversion efficiency of 22.64%. Under continuous AM 1.5G illumination without optical filters (xenon lamp, 65 °C, open-circuit conditions), the unencapsulated devices maintain over 90% of their initial efficiency after 192 h, providing a promising route to balance performance and durability.

## 1. Introduction

Narrow-bandgap tin–lead (Sn–Pb) mixed perovskite solar cells (PSCs), with an optimal bandgap of ~1.26 eV, are irreplaceable core components for high-efficiency all-perovskite tandem solar cells targeting efficiencies beyond the Shockley–Queisser (SQ) limit [1,2,3,4]. Beyond their critical role in tandem device architectures, Sn–Pb mixed PSCs also exhibit immense application potential in high-performance single-junction photovoltaic (PV) systems [5]. Specifically, the partial substitution of Pb with Sn enables precise bandgap tuning to approach the SQ-defined optimal bandgap for single-junction solar cells, while reducing the intrinsic lead toxicity of Pb-only perovskites, which is fully aligned with the sustainable development requirements of the PV industry [6,7,8].

Despite their exceptional theoretical optoelectronic performance, Sn–Pb mixed perovskites face far more intractable challenges than mature pure Pb-based counterparts, arising primarily from their intrinsic chemical instability and defect-driven performance degradation. The Sn^2+^ cation possesses a 4d^10^ 5s^2^ electron configuration, where the 5s lone-pair electrons impart a strong reducing nature, rendering Sn^2+^ extremely susceptible to oxidation to Sn^4+^ by ambient oxygen, photogenerated holes, and even iodine vacancies within the perovskite lattice [9,10,11]. This irreversible oxidation process not only compromises the structural integrity of the perovskite crystal lattice but also induces severe p-type self-doping. Consequently, the increased free-carrier density and proliferation of non-radiative recombination centers trigger rapid efficiency roll-off and severe device degradation [12,13,14]. Compounding this intrinsic instability, the lattice defects originating from Sn^2+^ oxidation can further trigger a series of additional detrimental issues, including phase segregation and accelerated ion migration, all of which exacerbate the operational stability of the devices under practical working conditions [15]. These degradation pathways are particularly pronounced at the surface of perovskite films, where the lower defect-formation energy facilitates the accumulation of undercoordinated metal cations (Sn^2+^/Pb^2+^) and halide vacancies. These interfacial defects act as reactive sites that accelerate Sn^2+^ oxidation. Meanwhile, they induce energy-level misalignment, create charge extraction barriers, impede efficient photogenerated-carrier transport, and ultimately deteriorate key device performance metrics, most notably the fill factor (FF) and short-circuit current density (J_SC_).

Against this backdrop, interfacial passivation engineering has emerged as a pivotal strategy to simultaneously address the efficiency and stability bottlenecks plaguing state-of-the-art Sn–Pb PSCs. To date, numerous interfacial passivation strategies have been reported for Sn–Pb perovskite systems. While molecular passivators—particularly Lewis bases such as ethylenediamine and ethylenediammonium diiodide—can partially alleviate these issues through coordination with undercoordinated metal cations, critical limitations remain [16,17]. Most conventional passivators function via a single modification mechanism (either chemical passivation or field-effect passivation) and thus fail to achieve comprehensive, multifunctional interfacial modification [18,19]. Amine- or ammonium-based salt passivators frequently introduce excess iodide species, which exacerbate ion migration and device hysteresis [20]. Furthermore, most of these molecular passivators lack sufficient coordination strength to durably suppress Sn^2+^ oxidation, while their bulky organic moieties tend to induce the formation of wide-bandgap low-dimensional perovskite phases [21,22]. Such phases introduce significant interfacial charge-transport barriers, ultimately offsetting the beneficial effects of passivation [23].

To overcome these interrelated limitations, we rationally designed morpholine acetate (MPAC) as a dual-functional interfacial passivator for Sn–Pb perovskite films. Compared to previously reported bifunctional passivators, the uniqueness of MPAC lies in its specific structural synergy: the strongly electron-donating acetate group ensures robust, localized coordination with undercoordinated metal cations without introducing excess halide ions, while the morpholine moiety forms a specific interfacial dipole layer that facilitates electron extraction without triggering the adverse segregation of wide-bandgap low-dimensional phases [24,25]. This synergistic modification enables regulated perovskite crystallization, long-term suppression of Sn^2+^ oxidation, optimized interfacial energy-level alignment, and enhanced carrier-extraction efficiency. Consequently, the MPAC-modified devices deliver a champion power conversion efficiency (PCE) of 22.64%, along with exceptional operational stability, retaining over 90% of their initial PCE after 192 h of rigorous accelerated-aging testing.

## 2. Experimental Section

### 2.1. Synthesis of MPAC

Morpholine (1.74 g, 20 mmol) and ethanol (40 mL) were added into a single-necked flask (100 mL) sequentially. Then, acetic acid (1.50 g, 25 mmol) was slowly added dropwise under ice-water-bath conditions, and the mixture was stirred gently for 3 h. After evaporating the solvent, the white product was washed with ethyl acetate three times to afford flake-like crystals (2.5 g, 85%). ^1^H NMR (500 MHz, CDCl_3_) δ: 9.44 (s, 2H), 3.78 (t, 4H, J = 5 MHz), 2.99 (t, 4H, J = 5 MHz), 1.97 (s, 3H); ^13^C NMR (126 MHz, CDCl_3_) δ: 177.67, 65.81, 44.32, 23.15. ^1^H NMR and ^13^C NMR can be found in Figure S1.

### 2.2. Device Fabrication of Tin–Lead PSCs

The pre-patterned indium tin oxide (ITO, 15 Ω/m^2^) substrates were sequentially cleaned via ultrasonication in diluted Micro-90 detergent, deionized water, acetone, and isopropanol for 15 min, respectively. Then, the cleaned ITO substrates were dealt with UV ozone for 30 min before use. A PEDOT:PSS layer was then spin-coated at 3000 rpm for 30 s and thermally annealed at 150 °C for 15 min. The substrates were cooled down to room temperature before use.

1.25 M Cs_0.17_FA_0.83_Pb_0.5_Sn_0.5_I_3_ precursor solution was prepared by dissolving FAI, CsI, PbI_2_, SnI_2_, SnF_2_, and GuaSCN in a DMF:DMSO mixed solvent (3:1, *v*:*v*). During dissolution, 20% tin powder was added under continuous stirring at 45 °C. The perovskite films were deposited by spin-coating the precursor solution onto the PEDOT:PSS film at 5000 rpm for 5 s, followed by immediate transfer to a vacuum chamber for 15 s of vacuum flash evaporation treatment, and then annealed at 100 °C for 20 min. Following the deposition of the perovskite active layer, 100 μL of MPAC solution (0.5 mg/mL in IPA) was spin-coated onto the film at 5000 rpm for 30 s without annealing. This concentration was selected as the optimal value based on a systematic screening of concentrations (0.5, 1.0, and 1.5 mg/mL) and the detailed photovoltaic parameters are summarized in Table S1 of the Supporting Information. The Sn–Pb perovskite films were fabricated in an N_2_-filled glovebox with O_2_ and H_2_O concentrations below 0.1 ppm at around 23 °C. The films were then cooled to room temperature prior to thermal evaporation. Sequentially, 25 nm C_60_, 5 nm BCP and an 80 nm silver electrode were evaporated under a high vacuum (<3 × 10^−6^ Torr).

## 3. Results and Discussion

**Figure 1:**
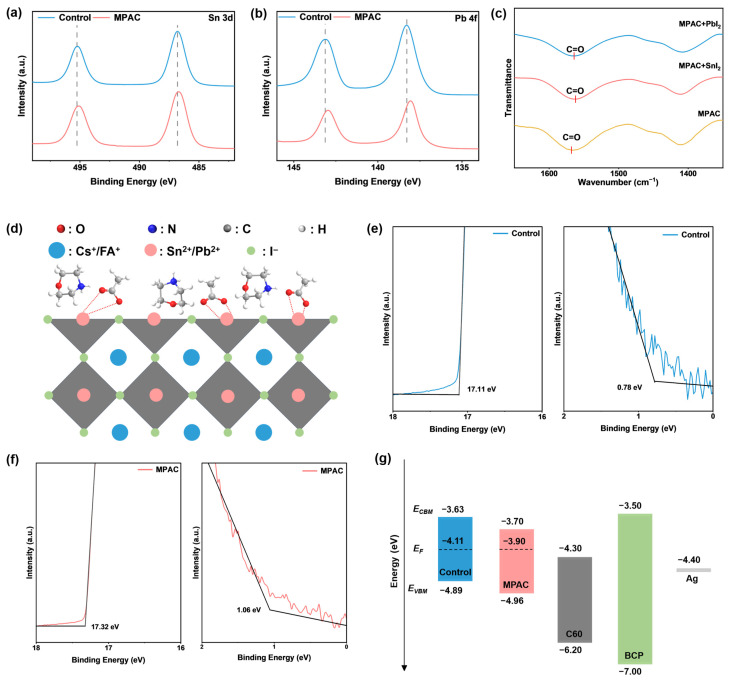
Chemical Structure of MPAC and Characterization of Its Coordination Interaction and Interfacial Energy-Level Modulation in Sn–Pb Perovskite Films. (**a**,**b**) XPS spectra of the pristine control and MPAC-modified Sn–Pb perovskite films. (**c**) FTIR spectra of pure MPAC powder, MPAC-PbI_2_ mixture, and MPAC-SnI_2_ mixture in the C=O stretching vibration region. (**d**) Schematic illustration of the bonding mode between the MPAC molecule and the Sn–Pb perovskite film surface. (**e**,**f**) UPS spectra of the high-binding energy cut-off region for the control and MPAC-modified perovskite films. (**g**) Schematic energy-level alignment of each functional layer in the control and MPAC-modified Sn–Pb perovskite solar cell devices.

We first employed X-ray photoelectron spectroscopy (XPS) to examine the chemical interaction between MPAC and the Sn–Pb perovskite surface. In the MPAC-modified film, the Sn 3d and Pb 4f core level peaks shifted toward lower binding energies compared to the control (Figure 1a,b). This shift provides direct evidence that the electron-rich groups in MPAC donate electrons to unoccupied orbitals of the surface metals, forming stable coordinate bonds [26,27]. Crucially, deconvolution of the Sn 3d_5/2_ spectra (utilizing a Shirley background, specific binding energy constraints, and full width at half maximum (FWHM) limits strictly constrained between 0.4 and 2.0 eV for Sn^2+^, Sn^4+^, and Sn^0^) revealed the specific contents of different Sn oxidation states. As shown in Figure S2a,b and summarized in Table S2, after optimization with MPAC, the Sn^4+^ content in the film dropped sharply from 21.4% to 13.1%, while the Sn^2+^ content concurrently increased from 59.2% to 75.6%. The presence of Sn^0^ detected in the films is likely generated by the disproportionation reaction of Sn^2+^ [10]. This highly reproducible shift in the Sn^4+^/Sn^2+^ ratio provides direct, quantitative evidence that MPAC successfully limits Sn^2+^ oxidation and suppresses p-type self-doping [28].

To theoretically elucidate the molecular origin of this electron donation, we performed electrostatic potential (ESP) calculations on the intact MPAC complex (Figure S3). The ESP map reveals a profound intramolecular charge separation, with the most concentrated negative potential strictly localized at the oxygen atoms of the acetate anion. This highly electron-rich nature suggests that the acetate group, particularly the carbonyl oxygen, should act as the primary nucleophilic site for donating lone-pair electrons to undercoordinated metal cations (Sn^2+^/Pb^2+^). Guided by this theoretical prediction, we turned to Fourier transform infrared (FTIR) spectroscopy (Figure 1c). The characteristic C=O stretching peak of pristine MPAC at 1568.2 cm^−1^ redshifted to 1562.1 cm^−1^ and 1564.0 cm^−1^ upon mixing with SnI_2_ and PbI_2_, respectively. This confirms that the carbonyl group of the acetate moiety acts as the primary anchoring site for Sn^2+^ and Pb^2+^ (Figure 1d). The more pronounced shift observed with SnI_2_ aligns with the stronger Lewis acidity of Sn^2+^, corroborating both our theoretical ESP predictions and XPS findings.

Having established the mechanism of chemical passivation, we next utilized Kelvin probe force microscopy (KPFM) to investigate the field-effect modulation (Figure S4). Upon MPAC modification, the average contact potential difference (CPD) of the perovskite film shifted positively from 237 mV to 402 mV. This substantial 165 mV shift is indicative of the formation of an interfacial dipole layer, which repels photogenerated holes and facilitates electron extraction [29,30,31]. To quantify how this dipole layer reshapes the energy landscape, we conducted ultraviolet photoelectron spectroscopy (UPS) (Figure 1e,f). The high-binding energy cut-off (E_cut-off_) shifted from 17.11 eV to 17.32 eV, corresponding to a 0.21 eV decrease in work function (W_F_). Concurrently, the valence band maximum (VBM) downshifted by 0.28 eV. At the perovskite/C_60_ interface, this specific energy-level realignment provides a dual physical benefit: the deeper VBM establishes a higher hole-blocking barrier that effectively limits hole back-transfer, while the reduced W_F_ facilitates electron extraction to the fullerene layer. By optimizing these interfacial energetics to suppress non-radiative recombination and minimize carrier loss, the MPAC treatment directly accounts for the substantial enhancements observed in both the open-circuit voltage (V_OC_, increasing from 0.811 V to 0.877 V) and FF (increasing from 78.54% to 80.46%) of the modified devices (Figure 1g) [32,33].

**Figure 2:**
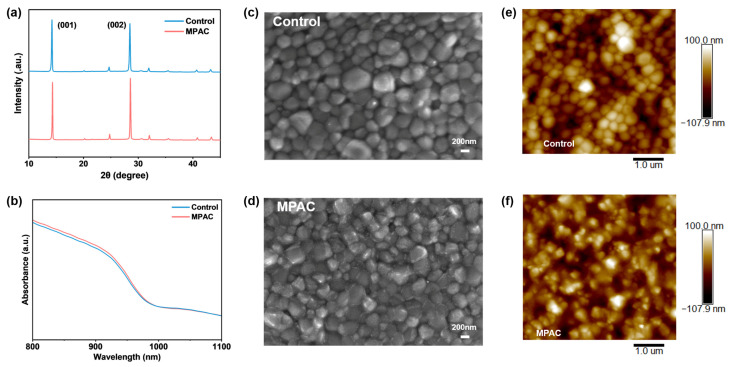
Effect of MPAC Modification on Crystallinity, Optical Absorption and Morphology of Sn–Pb Mixed Perovskite Films. (**a**) XRD patterns of the control and MPAC-modified perovskite films. (**b**) Ultraviolet–visible (UV-Vis) absorption spectra of the control and MPAC-modified perovskite films. (**c**,**d**) Top-view SEM images of the pristine control and MPAC-modified perovskite film. (**e**,**f**) AFM topography images of the control and MPAC-modified perovskite film.

While surface modifications are beneficial, it is vital that they do not perturb the underlying bulk properties. X-ray diffraction (XRD) was performed to investigate the effect of MPAC modification on the crystal structure of Sn–Pb perovskite films (Figure 2a). Both control and MPAC-modified films showed the characteristic (001) and (002) diffraction peaks at 14.2° and 28.4°, respectively, confirming that MPAC incorporation does not alter the intrinsic perovskite crystal structure [34]. No additional diffraction peaks corresponding to low-dimensional perovskite phases or PbI_2_/SnI_2_ phase segregation were detected in the MPAC-treated film, thereby avoiding the charge-transport barriers caused by adverse low-dimensional phases—a common drawback of conventional ammonium salt passivators [35]. Ultraviolet–visible (UV-Vis) absorption spectroscopy was used to evaluate the light-harvesting properties of the perovskite films (Figure 2b). The absorption edge of the MPAC-modified film remained unchanged at ~980 nm, corresponding to an optical bandgap of 1.26 eV, indicating that MPAC modification does not compromise the light-absorption capability of the perovskite and thus maintains the potential for a high J_SC_ of the device.

Scanning electron microscopy (SEM) was employed to characterize the surface morphology of the perovskite films (Figure 2c,d). The control film exhibited a polycrystalline morphology with abundant pinholes and small grains, while the MPAC-modified film showed a dense, pinhole-free microstructure with significantly reduced grain boundary defects. Statistical analysis (Figure S5) revealed that the average grain size of the perovskite film increased from 293.5 nm (control) to 347.2 nm after MPAC treatment. This grain enlargement suggests that the specific adsorption of MPAC molecules promotes more extensive secondary grain growth during annealing, likely due to their ability to minimize the surface free energy of crystal nuclei [36]. Similar mechanisms have been reported by Yang et al., where surface modifications were found to drive secondary grain growth and improve perovskite crystallinity through crystalline liquid-like behavior and surface energy reduction.

Atomic force microscopy (AFM) was further conducted to assess the surface roughness of the perovskite films (Figure 2e,f). The root-mean-square (RMS) roughness of the MPAC-modified film decreased to 28.6 nm from 31.3 nm for the control film. The reduced roughness not only reflects improved film quality with fewer morphological defects but also enables better interfacial contact between the perovskite and the electron transport layer (ETL), minimizing interfacial contact resistance and facilitating efficient charge-carrier extraction.

**Figure 3:**
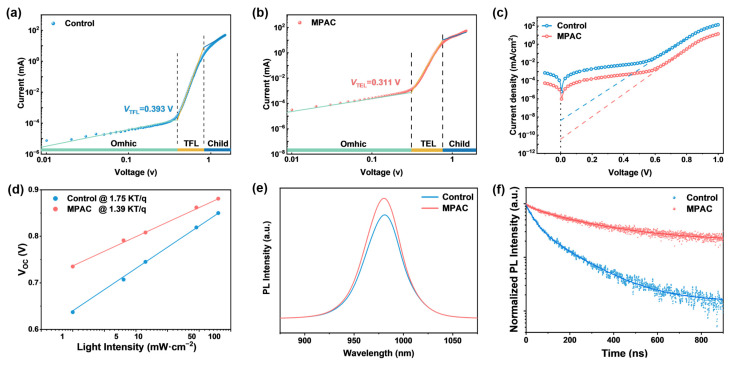
MPAC-Mediated Defect Passivation and Charge-Carrier Dynamics in Sn–Pb Perovskite Films. (**a**) SCLC J-V curves of the electron-only control device. (**b**) SCLC J-V curves of the electron-only MPAC-modified device. (**c**) Dark J-V characteristics of control and MPAC-modified devices. (**d**) V_OC_ versus light intensity for control and MPAC-modified devices. (**e**) Steady-state PL spectra of control and MPAC-modified films. (**f**) TRPL decay curves of control and MPAC-modified films.

To evaluate how these structural improvements translate into electronic quality, we quantified the trap-state density (N_trap_) via space-charge-limited current (SCLC) measurements (Figure 3a,b). The trap-filled limit voltage (V_TFL_) decreased from 0.393 V for the control device to 0.311 V for the MPAC-modified device, corresponding to a reduction in N_trap_ from 3.22 × 10^15^ cm^−3^ to 2.76 × 10^15^ cm^−3^. This significant drop in trap density directly demonstrates the effective defect passivation of MPAC in Sn–Pb perovskite films. Accordingly, dark J-V characteristics showed that the reverse saturation current density of the MPAC-modified device was reduced by nearly two orders of magnitude, from 4.07 × 10^−9^ mA/cm^2^ for the control to 3.55 × 10^−11^ mA/cm^2^ (Figure 3c). This substantial reduction confirms that defect-induced leakage current is effectively suppressed and that non-radiative recombination in the device is significantly inhibited by MPAC modification. The dependence of V_OC_ on light intensity was analyzed to reveal the dominant charge-carrier recombination mechanism in the devices (Figure 3d). The ideality factor (n_id_), a key indicator of the recombination pathway, decreased significantly from 1.75 for the control device to 1.39 for the MPAC-modified device, approaching unity. This result strongly signifies that defect-mediated Shockley–Read–Hall (SRH) non-radiative recombination is effectively suppressed after MPAC modification, consistent with the SCLC measurement results [37,38].

Steady-state photoluminescence (PL) and time-resolved PL (TRPL) spectroscopy were conducted to directly characterize the charge-carrier dynamics and non-radiative recombination loss in the perovskite films (Figure 3e,f). The MPAC-modified film exhibited a markedly enhanced PL intensity without a noticeable peak shift compared with the control film, indicating suppressed non-radiative recombination via defect passivation. As shown in Table S3, fitting of the TRPL decay curves showed that the average carrier lifetime was significantly prolonged from 131.58 ns for the control film to 281.45 ns for the MPAC-treated film, further confirming the effective inhibition of trap-assisted non-radiative recombination and optimized carrier dynamics enabled by MPAC modification [39]. Transient photocurrent (TPC) measurements were performed to investigate the effect of MPAC on interfacial charge-carrier extraction (Figure S6). The fitted charge extraction lifetime was reduced from 32 μs for the control film to 15 μs for the MPAC-modified film, clearly demonstrating that MPAC-induced field-effect passivation greatly facilitates interfacial charge-carrier extraction.

**Figure 4:**
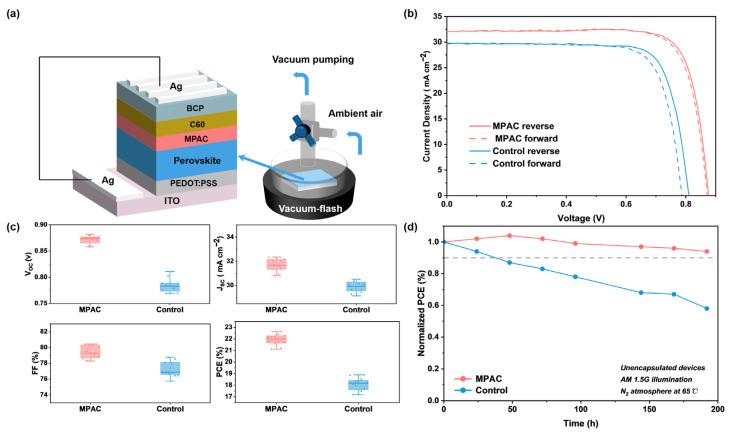
Photovoltaic Performance and Stability of MPAC-Modified Sn–Pb Perovskite Solar Cells. (**a**) Schematic of the inverted perovskite solar cell device architecture. Device configuration for fabricating perovskite films via the vacuum flash evaporation technique. (**b**) J-V curves of champion control and MPAC-modified devices under AM 1.5G illumination. (**c**) Statistical distribution of photovoltaic parameters for 12 independent MPAC-modified and control devices. (**d**) Long-term stability of unencapsulated control and MPAC-modified devices in a nitrogen atmosphere.

Building upon these parameters, we fabricated inverted Sn–Pb perovskite solar cells with a device structure of ITO/PEDOT:PSS/Perovskite/MPAC/C_60_/BCP/Ag (Figure 4a). Notably, the Sn–Pb perovskite film was prepared by using a vacuum-flash solution processing method. Under standard AM 1.5G illumination, the champion MPAC-modified device achieved a reverse-scan PCE of 22.64%, with the V_OC_ of 0.877 V, J_SC_ of 32.09 mA/cm^2^, and FF of 80.46%, significantly outperforming the pristine control device (PCE of 18.90%, V_OC_ of 0.811 V, J_SC_ of 29.66 mA/cm^2^, FF of 78.54%) (Figure 4b; detailed parameters are provided in Table S4). Importantly, MPAC modification significantly mitigates J-V hysteresis, reducing the hysteresis index from 4.39% to 1.55% through synergistic chemical and field-effect passivation. This suppression results from the immobilization of halide vacancies and optimized interfacial energetics, which collectively inhibit ion migration and charge accumulation at the perovskite interface [20]. Unlike conventional salt passivators, the halogen-free structure of MPAC ensures superior steady-state output without introducing additional mobile ions.

The statistical photovoltaic parameters of 12 independent MPAC-modified and without modification devices are plotted in Figure 4c. Quantitatively, the device performance showed substantial improvement after MPAC passivation, with the average PCE increasing from 18.01 ± 0.83% to 21.95 ± 0.70%. Specifically, MPAC-modified devices exhibited average values of V_OC_ of 0.874 ± 0.016 V, J_SC_ of 31.67 ± 0.85 mA/cm^2^, and FF of 79.29 ± 1.17%. In contrast, control devices displayed average values of V_OC_ of 0.783 ± 0.028 V, J_SC_ of 29.92 ± 0.78 mA/cm^2^, and FF of 76.86 ± 1.89% (Table S5). These results clearly demonstrate that the synergistic chemical and field-effect passivation of MPAC not only boosts efficiency but also significantly improves device reproducibility by suppressing non-radiative recombination and optimizing interfacial energy-level alignment.

We further evaluated the long-term operational stability of unencapsulated devices via a rigorous aging test performed under continuous full-spectrum AM 1.5 illumination without optical filters, at an equivalent temperature of 65 °C, with the devices maintained in an open-circuit state throughout the test period. It is worth noting that the open-circuit state is the most stringent test environment for PSCs: photogenerated carriers cannot be effectively exported to the external circuit, leading to severe carrier accumulation, which drastically exacerbates non-radiative recombination, Sn^2+^ oxidation, and ion migration, thereby posing a far greater challenge to device stability than conventional maximum power point tracking operation. As shown in Figure 4d, the MPAC-passivated device retained over 90% of its initial PCE after 192 h of aging, while the control device retained only 70% of its initial efficiency after 144 h, confirming the remarkable enhancement in long-term operational stability enabled by MPAC modification.

## 4. Conclusions

In conclusion, we report MPAC as a bifunctional interfacial passivator that enables synergistic chemical and field-effect passivation for narrow-bandgap Sn–Pb PSCs. For chemical passivation, XPS confirms that the acetate group of MPAC coordinates with undercoordinated Sn^2+^/Pb^2+^ cations, directly inhibiting spontaneous Sn^2+^ oxidation and reducing the Sn^4+^ content in perovskite films from 21.4% to 13.1%. For field-effect passivation, UPS verifies that the morpholine moiety induces a stable interfacial dipole layer, reducing the perovskite work function by 0.21 eV and downshifting the valence band maximum by 0.28 eV, thus optimizing the perovskite/C_60_ energy-level alignment for efficient charge extraction. This dual-function passivation effectively suppresses trap-assisted non-radiative recombination, with significantly reduced trap-state density and more than doubled carrier lifetime in the modified films. For the inverted Sn–Pb PSCs studied in this work, the MPAC-modified champion device achieves a PCE of 22.64%, markedly outperforming the 18.90% PCE of the control device. Benefiting from sustained Sn^2+^ oxidation inhibition and interfacial defect suppression, the unencapsulated MPAC-modified device retains over 90% of its initial PCE after 192 h of accelerated operational aging, while the control device retains only 70% of its initial efficiency after 144 h under identical test conditions. This work offers a validated reference for suppressing Sn^2+^ oxidation and optimizing interfacial carrier dynamics in narrow-bandgap Sn–Pb perovskite solar cells.

## Data Availability

The original contributions presented in this study are included in the article/Supplementary Information. Further inquiries can be directed to the corresponding authors.

## Supplementary Materials

The following supporting information can be downloaded at: https://www.mdpi.com/article/10.3390/ma19101914/s1, Table S1: Effect of MPAC concentrations on device performance; Table S2: Contents of Sn with different valence states in the films; Table S3: Fitting data of TRPL curves in Figure 3f based on double-exponential fitting; Table S4: Photovoltaic parameters of MPAC-treated and control devices; Table S5: Statistical table of photovoltaic parameters for 12 independent MPAC-modified and control devices. Figure S1: The 1H and 13C NMR of MPAC (500 MHz, CDCl3); Figure S2: XPS spectra of Sn 3d_5/2_ core level for (a) control and (b) MPAC-treated perovskite films; Figure S3: ESP mapping of the MPAC molecule; Figure S4: KPFM images of the pristine (a) and MPAC-treated perovskite films (b), while (c) and (d) show the corresponding contact potential difference along the white dashed line in the KPFM images; Figure S5: Grain sizes distribution of (a) control, (b) MPAC-treated perovskite films; Figure S6: TPC curves of MPAC-treated and control devices.
