# Supplementary material for: Synergistic Chemical and Field-Effect Passivation Inhibits Sn2+ Oxidation and Non-Radiative Recombination in Tin–Lead Perovskite Solar Cells
Source: Materials (Basel). 2026 May 7;19(10):1914. doi: 10.3390/ma19101914 (PMC13208191; doi:10.3390/ma19101914)
Supplement: Supplementary file 1 [file materials-19-01914-s001.zip › materials-4278681-supplementary.pdf]

Supplementary Information for

Synergistic Chemical and Field-Effect Passivation Inhibits Sn^{2+} Oxidation and Non-Radiative Recombination in Tin-Lead Perovskite Solar Cells

Jiahao Liu[1, †], Xucheng Wang[1, †], Pan Li[2], Huiyan Chen[2], Xing Tang[1], Weidong Lin[1], Ye Yuan[1, *], Xuehui Xu[2, *]

[1]State Key Laboratory of Advanced Technology for Materials Synthesis and Processing, Wuhan University of Technology, Wuhan 430070, China; 345201@whut.edu.cn (J.L.); wxc322223@whut.edu.cn (X.W.); 345253@whut.edu.cn (X.T.); 359286@whut.edu.cn (W.L.)

[2]State Key Laboratory of Modern Optical Instrumentation, Institute of Advanced Photonics, College of Optical Science and Engineering, Zhejiang University, Hangzhou, Zhejiang 310027, China;

22430084@zju.edu.cn (P.L.); 12330062@zju.edu.cn (H.C.)

[*]Correspondence: Corresponding author: Ye Yuan (fyyuanye@whut.edu.cn), Xuehui Xu (xuehui.xu@zju.edu.cn)

[†] These authors contribute equally to this work.

Material and synthesis

All the chemicals were purchased from commercial businesses and without further purification, including CsI (99.99%), PEDOT:PSS (Al 4083), fullerene-C_{60} (C_{60}, 99.5%) and 2,9-dimethyl-4,7-diphenyl-1,10-phenanthroline (BCP, 99%) were purchased from Xi'an Yuri Solar Co., Ltd. SnI_2 (99.999%), SnF_2 (99%) and Tin powder (99.99%) were bought from Thermo Scientific. Formamidinium iodide (FAI, 99.9%), PbI_2 (99.99%), guanidinium thiocyanate (GuaSCN, 99%) and morpholine (99%) were purchased from TCI. N,N-Dimethylformamide (DMF, 99.8%, anhydrous), dimethyl sulfoxide (DMSO, 99.9%, anhydrous) and 2-propanol (IPA, 99.9%, anhydrous) were obtained from Sigma-Aldrich. Acetic acid (AR) was purchased from Sinopharm Chemical Reagent Co., Ltd.

Characterizations

The ^{1}H NMR and ^{13}C NMR spectra were collected on a Bruker Avance III HD 500 MHz spectrometer (Bruker, Billerica, MA, USA) in $CDCl_3$. Current-density-voltage (J-V) characteristics were measured under AM 1.5G light (100mW/cm^2) using the xenon arc lamp of a Class A solar simulator (Newport, Irvine, CA, USA). Light intensity was calibrated using a Newport-calibrated mono Si diode. The Keithley 2400 source meter (Keithley Instruments, Cleveland, OH, USA) was used for J-V measurement. The devices were shielded with a shading mask with an aperture area of 0.0585 cm^2. The morphology of the samples was recorded by scanning electron microscopy (SEM, Ultra 55, Carl Zeiss, Oberkochen, Germany). Atomic force microscopy (AFM) and Kelvin probe force microscopy (KPFM) were performed using a Bruker MultiMode 8-HR

(Bruker, Santa Barbara, CA, USA). X-ray photoelectron spectroscopy (XPS) and Ultraviolet photoelectron spectroscopy (UPS) characterization were conducted by a Thermo Scientific Escalab 250Xi surface analysis system (Thermo Fisher Scientific, Waltham, MA, USA) equipped with a He discharge lamp (hv=21.22 eV) and a monochromatic Al-Kα X-ray gun (hv=1486.6 eV), respectively. X-ray diffraction (XRD) characterization was conducted on D2 Phaser instrument (Bruker, Karlsruhe, Germany) with a Cu Kα (a wavelength of 1.5418 Å) radiation. Fourier transform infrared spectrometer (FTIR) measurements were carried out using an Thermo Nicolet iS5 (Thermo Fisher Scientific, Waltham, MA, USA). Steady-state photoluminescence (PL) and time-resolved photoluminescence spectrum (TRPL) were conducted by Nikon C2+ Confocal System (Nikon, Tokyo, Japan) with a pulsed excitation laser of 515 nm. SCLC measurements were conducted using electron-only devices. Theoretical electrostatic potential (ESP) calculations were performed using Gaussian 16 software (Gaussian, Inc., Wallingford, CT, USA). The molecular models and surface charge distributions were visualized using GaussView (Version 6.0.16).

Table S1. Effect of MPAC concentrations on device performance

Concentrations (mg/mL)	V_{OC} (V)	J_{SC} (mA/cm^2)	FF (%)	PCE (%)
0.5	0.870 ± 0.012	31.32 ± 0.27	79.90 ± 1.17	21.77 ± 0.63
1.0	0.848 ± 0.009	29.67 ± 0.12	78.88 ± 0.81	19.85 ± 0.14
1.5	0.837 ± 0.003	28.23 ± 1.16	78.39 ± 1.88	18.52 ± 0.55

Table S2. Contents of Sn with different valence states in films

Samples	Sn^0 (%)	Sn^{2+} (%)	Sn^{4+} (%)
Control	19.4	59.2	21.4
MPAC	11.3	75.6	13.1

Table S3. Fitting data of TRPL curves in Figure 3f based on double exponential fitting.

Sample	A_1	τ_1 (ns)	A_2	τ_2	τ_{ave} (ns)
Control	54.83	35.55	38.75	161.49	131.58
MPAC	14.64	46.55	62.38	290.29	281.45

PL decay fitting curve is based on the bi-exponential decay equation:

$$I(t) = I_0 + A_1 exp\left(\frac{-t}{\tau_1}\right) + A_2 exp\left(\frac{-t}{\tau_2}\right) \quad (1)$$

Where τ_1 is the time constant for non-radiative recombination, and τ_2 is the time constant for radiative recombination. A_1 and A_2 are the amplitudes corresponding to non-radiative recombination and radiative recombination, respectively.

Table S4. Photovoltaic parameters of MPAC treated and control devices.

Devices	Scan Direction	V_{OC} (V)	J_{SC} (mA/cm^2)	FF (%)	PCE (%)
MPAC	Reverse	0.877	32.09	80.46	22.64
	Forward	0.873	32.13	79.45	22.29
Control	Reverse	0.811	29.66	78.54	18.90
	Forward	0.788	29.83	76.88	18.07

Table S5. Statistical table of photovoltaic parameters for 12 independent MPAC-modified and control devices.

Devices	V_{OC} (V)	J_{SC} (mA/cm^2)	FF (%)	PCE (%)
MPAC	0.874 ± 0.016	31.67 ± 0.85	79.29 ± 1.17	21.95 ± 0.70
Control	0.783 ± 0.028	29.92 ± 0.78	76.86 ± 1.89	18.01 ± 0.83

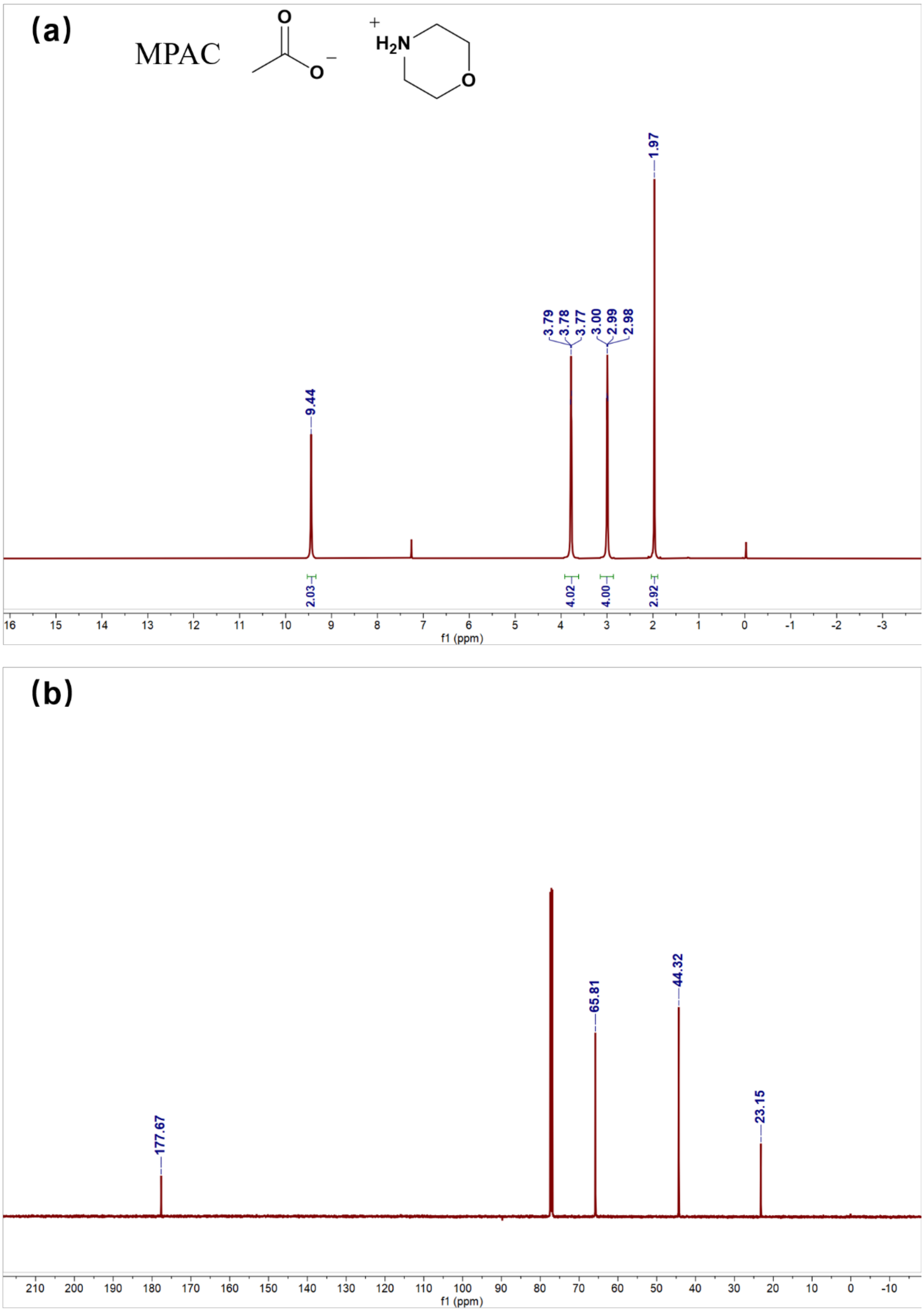

Figure S1. The ^{1}H and ^{13}C NMR of MPAC (500 MHz, $CDCl_3$).

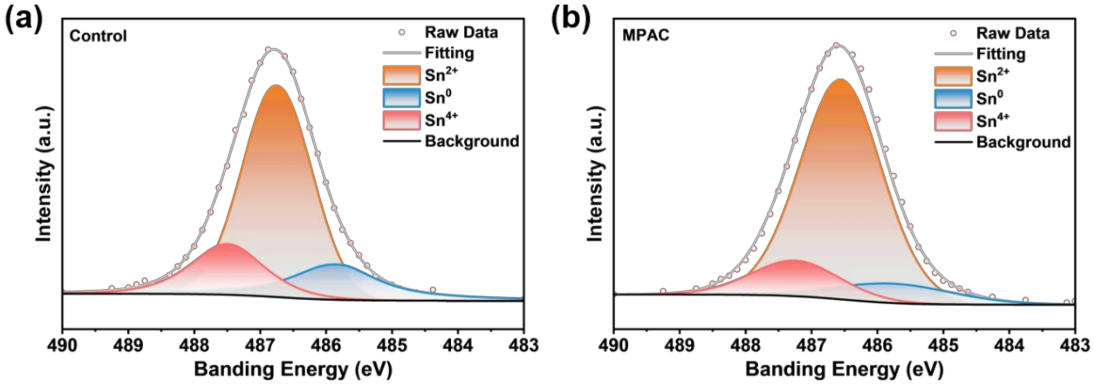

Figure S2. XPS spectra of Sn $3d_{5/2}$ core level for (a) control and (b) MPAC treated perovskite films.

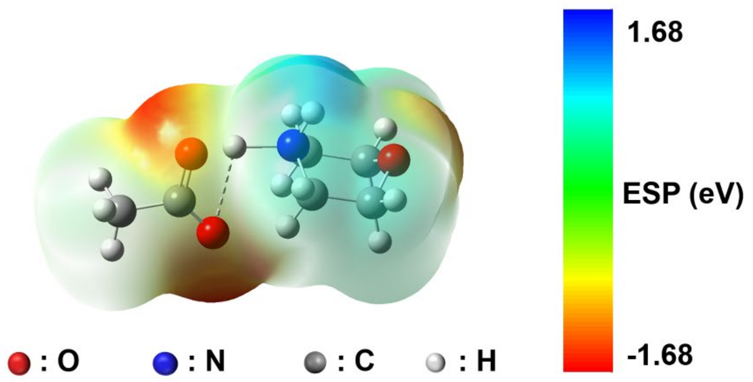

Figure S3. ESP mapping of the MPAC molecule.

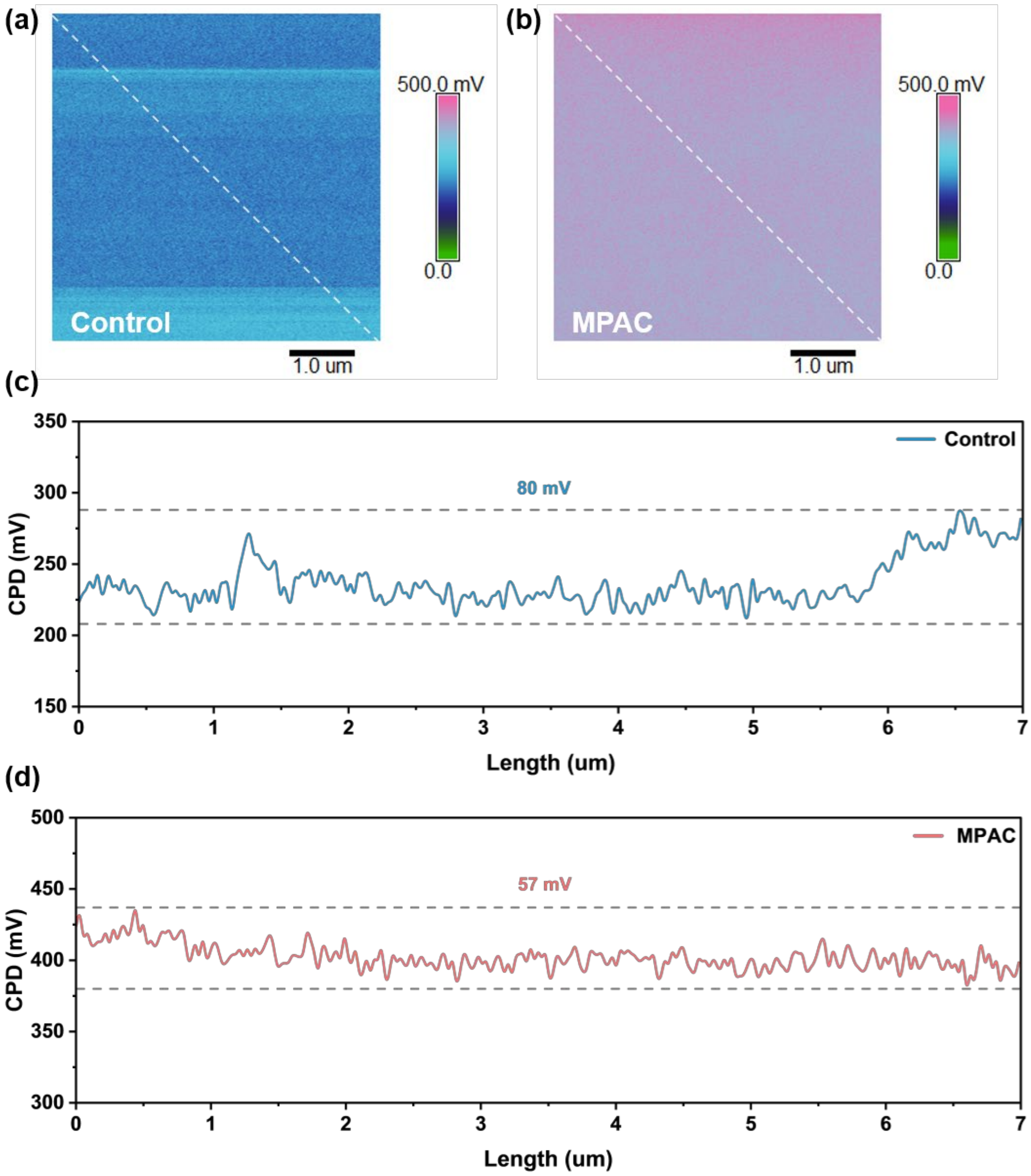

Figure S4. KPFM image of the pristine (a), and MPAC treated perovskite films (b). (c) and (d) show the corresponding contact potential difference along the white dashed line in the KPFM images.

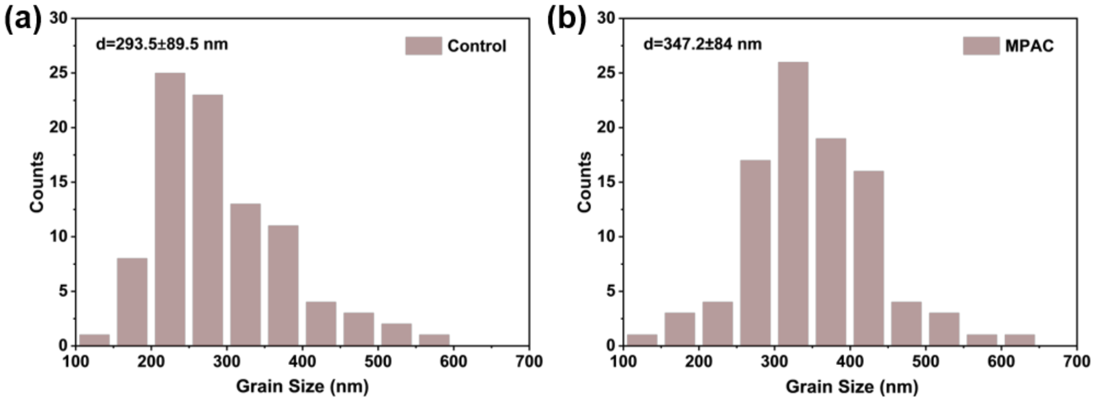

Figure S5. Grain sizes distribution of (a) control, (b) MPAC treated perovskite films.

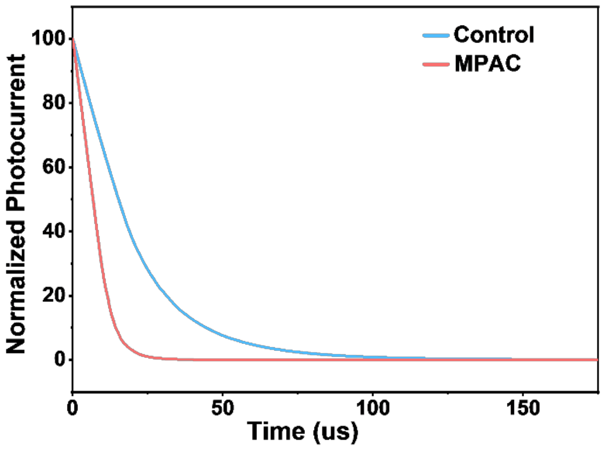

Figure S6. TPC curves of MPAC treated and control devices.
